# Integrated Chromatin Accessibility and Transcriptome Landscapes of Doxorubicin-Resistant Breast Cancer Cells

**DOI:** 10.3389/fcell.2021.708066

**Published:** 2021-07-30

**Authors:** Xuelong Wang, Jizhou Yan, Baiyong Shen, Gang Wei

**Affiliations:** ^1^Department of General Surgery, Pancreatic Disease Center, Ruijin Hospital, Shanghai JiaoTong University School of Medicine, Shanghai, China; ^2^CAS Key Laboratory of Computational Biology, Shanghai Institute of Nutrition and Health, University of Chinese Academy of Sciences, Chinese Academy of Sciences, Shanghai, China; ^3^Research Institute of Pancreatic Diseases, Shanghai JiaoTong University School of Medicine, Shanghai, China; ^4^Department of Developmental Biology, Institute for Marine Biosystem and Neurosciences, Shanghai Ocean University, Shanghai, China; ^5^Institute of Translational Medicine, Shanghai JiaoTong University, Shanghai, China

**Keywords:** doxorubicin resistance, chromatin accessibility, breast cancer, differentially accessible regions, transcription factor

## Abstract

**Background:**

Doxorubicin is one of the most effective chemotherapeutic drugs for breast cancer while its common drug resistance leads to poor patient prognosis and survival. Growing evidence indicate dynamically reorganized chromatin allows rapid access of the gene regulatory machinery to open genomic regions facilitating subsequent gene expression through direct transcription factor (TF) activation and regulatory element binding.

**Methods:**

To better understand the regulatory network underlying doxorubicin resistance in breast cancer cells, we explored the systematic alterations of chromatin accessibility and gene expression by the assay for transposase-accessible chromatin using sequencing (ATAC-seq) in combination with RNA sequencing, followed by integrative analysis to identify potential regulators and their targets associated with differentially accessible regions (DARs) in doxorubicin-resistant MCF7 (MCF7-DR) cells.

**Results:**

A total of 3,963 differentially expressed genes (DEGs) related to doxorubicin resistance were identified, including dramatically up-regulated *MT1E*, *GSTP1*, *LDHB*, significantly down-regulated *TFF1*, *UBB*, *DSCAM-AS1*, and histone-modifying enzyme coding genes *HDAC2*, *EZH2*, *PRMT5*, etc. By integrating with transcriptomic datasets, we identified 18,228 DARs in MCF7-DR cells compared to control, which were positively correlated with their nearest DEGs (*r* = 0.6). There were 11,686 increased chromatin-accessible regions, which were enriched in up-regulated genes related to diverse KEGG pathways, such as the cell cycle, regulation of actin cytoskeleton, signaling pathways of MAPK, PI3K/Akt and Hippo, which play essential roles in regulating cell apoptosis, proliferation, metabolism, and inflammatory responses. The 6,542 decreased chromatin-accessible regions were identified for the declined doxorubicin-associated biological processes, for instance, endocrine and insulin resistance, central carbon metabolism, signaling pathways of TGF-beta and P53. Combining data from TCGA, analyses of the DAR sequences associated with the DNA-binding motifs of significantly enriched TF families including AP-1, TEAD and FOX, indicated that the loss-function of FOXA1 might play a critical role in doxorubicin-resistant breast cancer cells (DOX-R BCCs).

**Conclusion:**

These data exhibit the non-genetic landscape of chromatin accessibility and transcript levels in the DOX-R BCCs, and provide clear insights and resources for the detection of critical TFs and potential *cis*-regulatory elements-based putative therapeutic targets.

## Introduction

Breast cancer has the highest morbidity and mortality rates of all cancers in the female population, and its incidence has gradually increased over the past few decades (Sung et al., 2021). Treatment options for breast cancer are dependent on the specific biological characteristics and genetic heterogeneity of the tumor. Despite significant progress has been made regarding treatment options for patients in recent years, drug resistance in cancer cells is one of the major obstacles for effective chemotherapy to treat cancer, which causes treatment failure in almost 90% of patients with metastatic cancer (Hammond et al., 2016). With regard to breast cancer treatment, although doxorubicin, known as an anthracycline antibiotic, is currently considered to be one of the most effective agents, the resistance to doxorubicin is still a major problem in clinical practice and leads to an unsuccessful outcome in many patients. Overcoming doxorubicin resistance would represent a major advance in effective breast cancer management.

Doxorubicin induces cell death and cell growth arrest primarily by inhibition of the topoisomerase II activity, DNA intercalation and production of free radicals, leading to apoptosis, autophagy, senescence and necrosis of the fast-growing cancer cells (Burgess et al., 2008; Meredith and Dass, 2016). In spite of the numerous genes, non-coding RNA and signaling pathways associated with doxorubicin resistance in breast cancer have been identified (Tormo et al., 2019; Ciocan-Cartita et al., 2020; Tanaka et al., 2020), the epigenetic regulatory mechanism, especially the role of chromatin-mediated processes in the regulation of gene expression, has not been investigated yet. Therefore, exploring the regulatory elements of doxorubicin-resistant genes and their corresponding transcription factors (TFs) in breast cancer is critically important for the elucidation of the disease process.

Eukaryotic genomes, as is well known, are extremely compacted in chromatin, a complex of DNA and proteins, and approximately 1% of the genome is composed of accessible elements, such as promoters, enhancers, and other regulatory sequences (Bickmore and van Steensel, 2013). Chromatin structure is dynamically shaped in a cell-specific manner, and the open chromatin regions allow TFs and other regulators of transcription to access *cis*-regulatory elements and activate gene expression, while closed chromatin regions impede the accessibility of promoters and enhancers to transcription machinery components leading to gene silencing (Klemm et al., 2019). Based on this principle, the genome-wide profiling of chromatin accessibility can be used to identify candidate regulatory elements associated with specific transcriptional and epigenetic signatures (Buenrostro et al., 2013). Recent advances in epigenomic profiling have enabled the development of ATAC-seq, which is a reliable tool for the generation of high-resolution chromatin accessibility landscape and direct recognition and sensitive detection of open chromatin regions (Buenrostro et al., 2013), both in bulk and at the single-cell level (Cao et al., 2018; Lareau et al., 2019). Depending on effective enzymatic cleavage and transposition of Tn5, being much faster and more sensitive, ATAC-seq has been widely applied in diverse development and disease-associated transitions to identify and localize epigenetic landscapes and regulators that drive disease pathogenesis (Corces et al., 2018; Wang et al., 2018; Xie et al., 2020).

In this study, we characterized the landscapes and relationship of chromatin accessibility and gene transcription in doxorubicin-resistant MCF7 cells, using ATAC-seq coupled with the analysis of RNA-seq data. The analysis of differentially accessible regions (DARs) allowed us to identify the regulatory DNA sequences, putative TFs, and landscape of TF binding events that may be responsible for these changes.

## Materials and Methods

### Cell Lines and Cell Culture

The MCF7 and doxorubicin-resistant MCF7 (MCF7-DR) cell lines originally obtained from the Cell Bank of the Type Culture Collection of the Chinese Academy of Sciences Committee (Shanghai, China), were kindly provided by the key laboratory of breast cancer in Shanghai, Fudan University. All cells were cultured in DMEM medium (KEL Biotech, China), supplemented with 2 mM L-glutamine (Gibco, United States), 10% fetal bovine serum (KEL Biotech, China), 0.1 M sodium pyruvate (Gibco, United States), 50 units/mL penicillin and 50 μg/mL streptomycin (Sangon Biotech, China). The MCF7 and MCF7-DR cells were grown in 10-cm culture dishes at 37°C in a humidified 5% CO_2_ atmosphere, and the medium was renewed every 2–3 days. Whereas parental MCF7 cells were grown in a drug-free medium, resistant-derived cells were maintained in doxorubicin hydrochloride (MedChem Express, United States) at a concentration of 1 μM. Doxorubicin hydrochloride was stored at a concentration of 2 mM (2,000×) in DMSO (Sigma-Aldrich, United States) at −20°C.

Before any further experiments, the MCF7-DR cells were maintained in doxorubicin free medium for 3 days. Parental MCF7 cells were cultured in parallel with the MCF7-DR cells for comparison.

### RNA Library Construction and Sequencing (RNA-seq)

Total RNA was isolated from MCF7 and MCF7-DR cells using TRIzol Reagent (Invitrogen, United States) and purified using RNeasy Mini Kit (QIAGEN, Germany). Agilent Bioanalyzer 2100 was used to assess RNA integrity. The RNA-seq library preparation from 1 μg of total RNA was performed with TruSeq RNA Sample Prep Kit (Illumina, United States) according to the manufacturer’s instructions. Finally, PCR products were purified to remove adaptor dimers with VAHTS DNA Clean Beads (Vazyme Biotech, China). Library quantity was assessed using a Qubit 2.0 Fluorometer (Invitrogen, United States). 150 bp pair-end sequencing was performed on the Illumina HiSeq X Ten platform (Novogene Biotech, China).

### Assay for Transposase-Accessible Chromatin With High-Throughput Sequencing (ATAC-seq)

The ATAC-seq libraries were prepared as previously described with minor modifications (Buenrostro et al., 2013; Wu et al., 2016). Briefly, a total of 50,000 fresh cells were washed twice with 100 μl of cold PBS (KEL Biotech, China) and resuspended in 100 μl of lysis buffer [10 mM Tris–HCl (pH 7.4), 10 mM NaCl, 0.1% IGEPAL CA-630 (Sigma-Aldrich, United States) and 3 mM MgCl_2_] for 10 min on ice to prepare the nuclei. Immediately after lysis, the suspension of nuclei was spun at 500 g at 4°C for 5 min to remove the supernatant. Nuclei were then incubated with the 50 μl of Tn5 transposition reaction mix (DIATRE Biotech, China) at 37°C for 30 min. The stop buffer was then added directly into the reaction to end the tagmentation. DNA was purified using the QIAquick PCR Purification Kit (QIAGEN, Germany). PCR was performed to amplify the library for 12 cycles using the following PCR conditions: 72°C for 3 min; 98°C for 30 s; and thermocycling at 98°C for 15 s, 60°C for 30 s and 72°C for 3 min; following by 72°C 5 min. Libraries were purified with VAHTS DNA Clean Beads (Vazyme Biotech, China) to remove contaminating primer dimmers. Library quantity was checked using Qubit 2.0 Fluorimeter (Invitrogen, United States). Last, all libraries were sequenced on the Illumina HiSeq X Ten with 150 bp paired-end reads (Novogene Biotech, China).

### RNA-seq Data Processing

FASTQ files were evaluated for quality control using FastQC^1^. The sequences were aligned to the human genome build hg19 using STAR (Dobin et al., 2013). The RNA expression of a gene was quantified by FPKM (fragments per kilobase million reads) based on RefSeq gene annotation using Cuffdiff (Trapnell et al., 2012). Differential expression genes (DEGs) were filtered by a *q* value < 0.05, the absolute value of fold change (FC) > 1.5 and the average FPKM > 10 in at least one of the two groups. The “ggplot2” (Wickham, 2009) in R and deepTools 2.0 (Ramirez et al., 2014) were used for data normalization and visualization. The aligned BAM files were converted to bigWig format and normalized by reads per kilobase million (RPKM) with the “bamCoverage” from deepTools 2.0 (Ramirez et al., 2014).

### ATAC-seq Data Processing

The quality of sequenced ATAC-seq data was evaluated with FastQC (see text footnote 1). All sequencing data were mapped onto the hg19 human genome assembly using Bowtie2 (Langmead and Salzberg, 2012) with parameter –X 2000. SAMtools (Li et al., 2009) was used to remove duplicate reads, only non-duplicate reads kept in the BAM format were used for the subsequent analysis. Peak calling on nucleosome-free reads was performed by MACS2 (Zhang et al., 2008) using the module ‘‘callpeak’’ with the x following parameters --shift --100 --extsize 200. Picard Tools (v.2.2.4^2^) and ggplot2 (Wickham, 2009) were used to analyze and plot the distribution of paired-end sequencing fragment sizes. For data normalization and visualization, the BAM files were converted to the bigWig format using the “bamCoverage” (Ramirez et al., 2014) with RPKM. The heatmaps and average profiles were also generated using the “plotHeatmap” and “plotProfile” scripts in deepTools (Ramirez et al., 2014). To annotate the location of ATAC-Seq peaks in terms of important genomic features, we assigned their BED files to promoter-TSS (by default defined from −1 Kb to +100 bp of transcription start site), TTS (by default defined from −100 bp to +1 Kb of transcription termination site), intron, intergenic, exon, etc. using the annotatePeaks.pl script in HOMER (v4.10) (Heinz et al., 2010) with default parameters.

### Identification of Differentially Accessible Regions

To assess the chromatin accessibility changes on a genome-wide scale, deepTools (Ramirez et al., 2014) was used to compute the average scores (RPKM normalized values) for transposase hypersensitive sites (THSs) in each sample. It was defined as hyper-accessible regions (hyper) associated with increased ATAC-seq signals if the regions showed the average fold change > 1.5 in MCF7-DR cells compared to parental MCF7 cells. On the contrary, the hypo-accessible regions (hypo) associated with decreased ATAC-seq signal was defined if the regions showed average fold change > 1.5 in parental MCF7 cells compared to MCF7-DR cells. The heatmaps and average profiles of regions expanded to ±1.5 Kb surrounding the DARs center were generated using the “plotHeatmap” and “plotProfile” functions in deepTools (Ramirez et al., 2014).

### Correlation Analysis of DARs and DEGs

Annotation of DARs to genomic features was performed using the annotatePeaks.pl function in HOMER (Heinz et al., 2010). The DARs neighboring genes (distance to TSS < 100 Kb) were then overlapped with DEGs identified from RNA-seq. Fold changes of DARs and their nearest DEGs were used to calculate the Pearson’s correlation coefficient (PCC) and *P* value in R. Venn diagrams, boxplots and scatter plots were produced using “Venn Diagram” (Chen and Boutros, 2011) and “ggplot2” (Wickham, 2009) R packages.

### Enrichment Analysis of GO Terms and KEGG Pathways

The clusterProfiler (Yu et al., 2012) was used to analyze GO terms and KEGG pathways, which is an R package for functional classification and enrichment of gene clusters using the hypergeometric distribution. The enriched terms and pathways with *P* value < 0.05 were considered significant. A Cytoscape network analysis^3^ was conducted to investigate links between significantly enriched top 20 KEGG pathways and key DEGs associated with MCF7-DR cells.

### Transcription Factor Motif Enrichment and Occurrences Analysis

For the motif enrichment analysis of DARs, BEDTools (v.2.25.0) (Quinlan, 2014) was employed to obtain the peak summits overlapping with DARs, then we used findMotifsGenome (Heinz et al., 2010) to discover known motif surrounding ATAC-seq peak summits and to identify binding sites for a certain TF with the parameter –size −200,200. Motifs were only kept if the *P* value was < 0.01. The HOMER (v4.10) (Heinz et al., 2010) was used to identify the nearest genes of the top 50 TFs and analyze the occurrence probability of a certain TF using a 1 Kb window flanking the peak summits.

### PPI Network Construction Using STRING

Based on RNA-seq and ATAC-seq data, differentially expressed transcription factors (DETFs) positively correlated with DARs were analyzed using the STRING database to identify groups of TFs that have known and predicted protein-protein associations (Szklarczyk et al., 2017). The network was subdivided into differentially colored nodes by the Markov cluster algorithm (MCL) with the default inflation parameter 3.0. The co-expression scores between proteins were calculated based on RNA expression patterns, and protein co-regulation provided by ProteomeHD (Kustatscher et al., 2019).

### TCGA-BRCA Data Analysis

Based on the genomic and clinical data from the Cancer Genome Atlas Breast Invasive Carcinoma (TCGA-BRCA), the breast cancer samples were divided into two subgroups based on whether the Doxorubicin (a.k.a. Doxorubicin) was used alone, namely the doxorubicin alone group (Dox^+^; *n* = 300) and the other group (Dox^–^; *n* = 762). The Kaplan–Meier survival curves and log-rank test were performed by utilizing the “survival” and “survminer” packages in R to analyze survival differences for critical DEGs. The mRNA expression levels of representative genes were compared between the Dox^+^ and Dox^–^ subgroups using the Wilcoxon signed rank test in R software (version 4.0.5). Pearson’s correlation coefficients and *P* values were also calculated to assess the associations between critical genes using the “Hmisc” R package. The *P* values less than 0.05 were regarded as statistically significant.

### Statistical Analysis and Data Visualization

If not specified, R platform^4^ was used to compute statistics and generate plots throughout this manuscript. Student’s *t*-test, Pearson’s correlation coefficient, Wilcoxon signed rank test was used to assess the significance. Fold change, *P* values and false discovery rate (FDR) were calculated in analysis. Significant differences for all quantitative data were considered when ^∗^*P* < 0.05, ^∗∗^*P* < 0.01, ^∗∗∗^*P* < 0.001, and ^****^P < 0.0001. The genomic signal tracks were extracted and visualized using the WashU Epigenome Browser^5^.

## Results

### Characterization of Critical Genes, Functional and Pathway Enrichment Analyses Associated With Acquired Resistance to Doxorubicin

**FIGURE 1:**
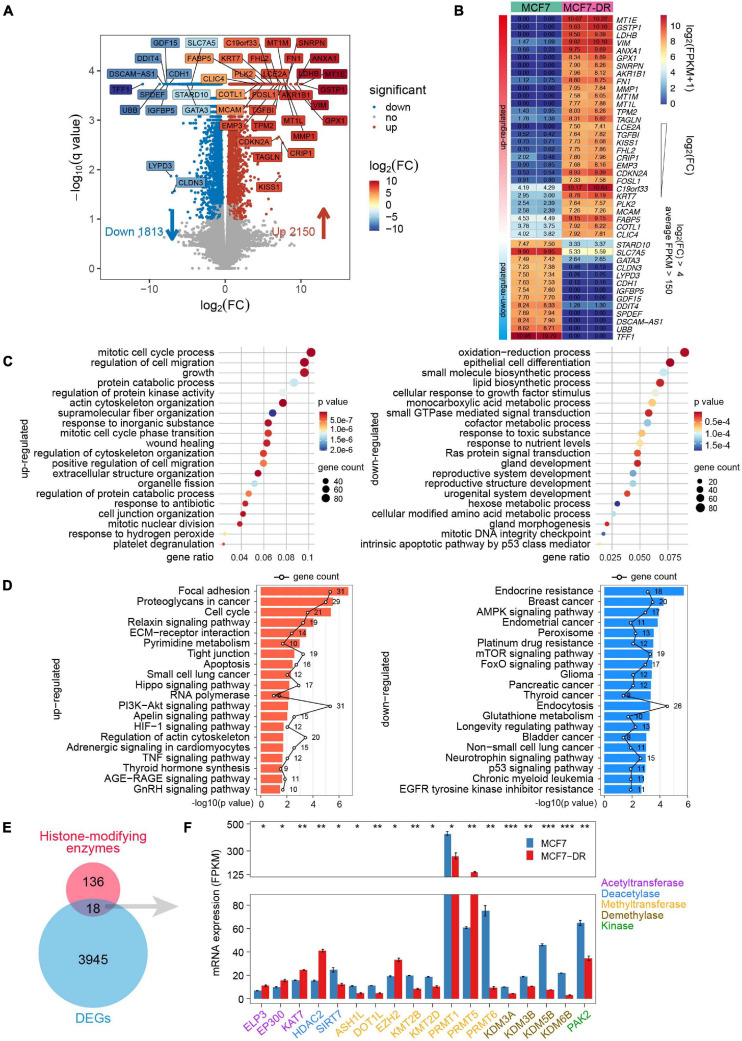
Identification of key genes and functional characteristics analysis. **(A)** Volcano plot showing the transcript levels of differentially expressed genes (DEGs) between the parental and doxorubicin-resistant MCF7 cells. Significantly up-regulated 2,150 genes are shown as red dots, whereas 1,813 down-regulated genes are shown as blue dots. Filtered by *q* value < 0.05, absolute log_2_(FC) > 4 and average FPKM > 150, 42 significantly up- and down-regulated genes are highlighted in different colors according to log_2_(FC). **(B)** Heatmap of 42 key genes labeled in the volcano plot (A), which are listed in a descending order based on log_2_(FC). The numeric values are log_2_(FPKM + 1). **(C)** Bubble plot showing GO enrichment analysis of DEGs. The top 20 GO terms of biological processes significantly enriched by up-regulated (left) and down-regulated (right) genes. **(D)** Bar plot showing top 20 KEGG pathways significantly enriched by up-regulated (left) and down-regulated (right) genes, respectively. The polygonal chain in black shows the gene count related to each GO term. **(E)** Venn diagram showing the overlap between the known histone-modifying enzymes and DEGs. **(F)** Bar plot indicating average expression levels (FPKM) of the 18 histone-modifying enzymes identified in panel **(E)**, including histone acetyltransferase (purple), deacetylase (blue), methyltransferase (orange), demethylase (brown) and kinase (green). The *P* values were calculated using Student’s *t* test in R. *P < 0.05, **P < 0.01, and ***P < 0.001.

To investigate potential genes associated with the resistance to doxorubicin in MCF7 breast cancer cells, RNA-seq was performed with two biological replicates for each sample (Supplementary Figures 1A–C). A total of 3,963 differential expression genes (DEGs) were identified, among which 2,150 genes were up-regulated and 1,813 genes were down-regulated (Figure 1A, Supplementary Figures 1D,E, and Supplementary Tables 1, 2). Furthermore, to reveal crucial candidate genes associated with doxorubicin resistance, filtered by log_2_(fold change) > 4, average FPKM > 150 in at least one group, we obtained 42 critical DEGs associated with doxorubicin resistance, including dramatically up-regulated metallothionein 1 family (*MT1E*, *MT1M*, and *MT1L*), *GSTP1* (glutathione S-transferase pi 1), *LDHB* (lactate dehydrogenase B), and significantly down-regulated *TFF1* (trefoil factor 1), *UBB* (ubiquitin B), *DSCAM-AS1* (DSCAM Antisense RNA 1).

GO analysis was applied to elucidate biological processes, which indicated that the up-regulated DEGs were mainly involved in the mitotic cell cycle process, cytoskeleton organization, regulation of protein kinase activity. Meanwhile, the down-regulated DEGs were significantly enriched in epithelial cell differentiation, oxidation-reduction process, lipid biosynthetic process and Ras protein signal transduction (Figure 1C). In addition, the KEGG pathway analysis revealed that the up-regulated DEGs were mainly enriched in focal adhesion, cell cycle, Hippo and HIF-1 signaling pathways. The down-regulated DEGs were enriched in endocrine and platinum resistance, breast cancer, FoxO and p53 signaling pathways (Figure 1D).

Based on RNA-seq data, we identified 18 differentially expressed known histone-modifying enzymes, which were involved in epigenetic control of critical cellular functions and post-translational modification of histone and non-histone substrates (Stillman, 2018; Morgan and Shilatifard, 2020; Figure 1E). The results revealed that several genes coding for histone acetyltransferases such as *ELP3*, *EP300* and *KAT7*, were significantly up-regulated; Except for *EZH2* and *PRMT5*, most of the differentially expressed histone methyltransferase and demethylase genes were down-regulated (Figure 1F).

### Genome-Wide Changes in Chromatin Accessibility

**FIGURE 2:**
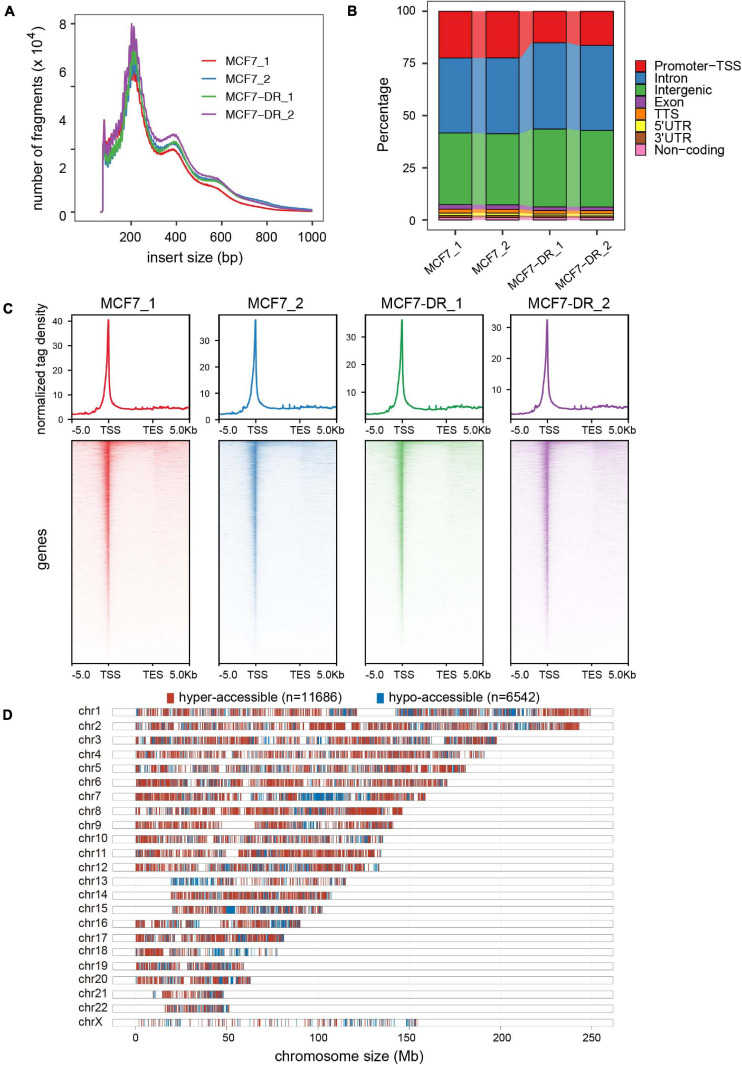
Genome-wide landscape of chromatin accessibility in MCF7 andMCF7-DR cells. **(A)** Distribution of ATAC-seq fragment size ineach sample. Clear modulation of signal is evident for mono-, di- and tri-nucleosomes. **(B)** Annotation of ATAC-seq peaks to genomic features: exon, intergenic regions, introns, 3′ UTR, 5′ UTR, promoters-TSS, TES and no-coding regions. Peak summit located within upstream –1,000 bp to + 100 bp downstream of the TSS is determined to be “promoter-TSS.” **(C)** The average read density (top) and heatmaps (bottom) indicating ATAC-seq signal across a genomic window of upstream –5 Kb to +5 Kb downstream of the TES. **(D)** Distribution of hyper- (red) and hypo-accessible (blue) regions over chromosomes in MCF7-DR cells compared to MCF7.

Assay for transposase-accessible chromatin with high-throughput sequencing (ATAC-seq) is the most current method for probing accessible chromatin regions, based on the ability of hyperactive Tn5 transposase to insert Illumina sequencing adaptors into active regulatory regions (Buenrostro et al., 2013; Wu et al., 2016; Li et al., 2017). To generate ATAC-seq libraries, we employed parental and doxorubicin-resistant MCF7 breast cancer cells with two independent biological replicates for each group (Supplementary Table 3). The chromatin was fragmented into mono-, di- and tri-nucleosome patterns and the similar distribution of fragment sizes indicates that chromatin is accessible to transposase to the same degree in all samples (Figure 2A). Genome-wide analysis of THSs distribution over functional genomic elements revealed that the promoter-TSS, introns and intergenic regions were preferentially (more than 90%) accessible to Tn5 transposase (Figure 2B). Moreover, our results conform to the pattern reported in previous studies in multiple organisms by showing that ATAC-seq signal was significantly enriched at ±1 Kb region surrounding TSS (Figure 2C), which suggests that the majority of cis-regulatory regions were located in the vicinity of gene core promoters and that open chromatin at THSs is predictive of active transcription (Buenrostro et al., 2013; Doganli et al., 2017; Lu et al., 2017; Bozek et al., 2019).

**FIGURE 3:**
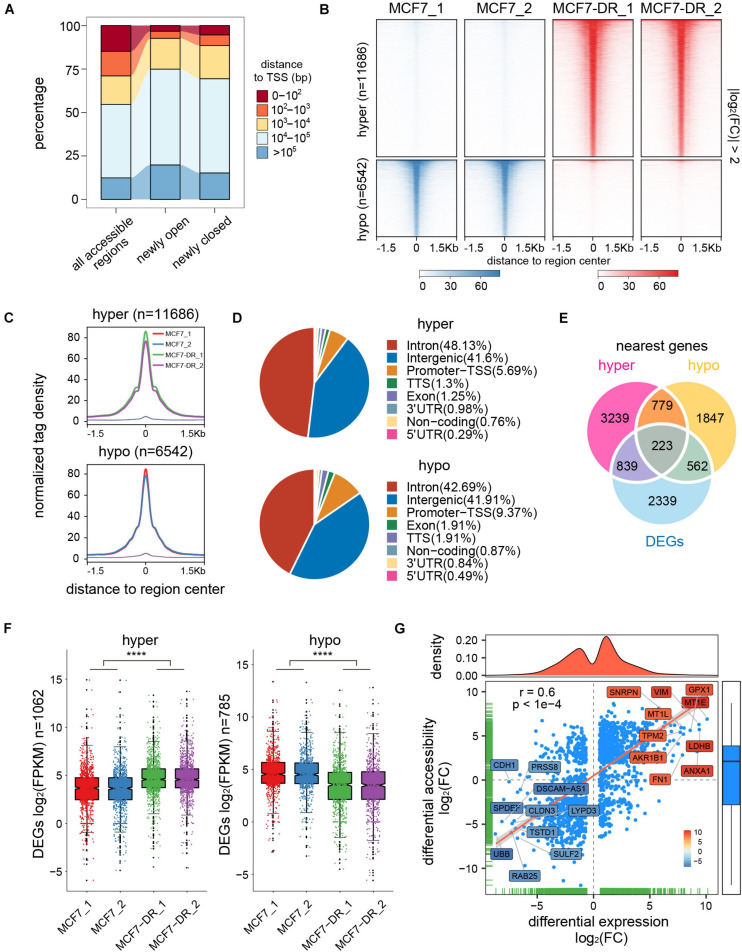
Identification and analysis of differentially accessible regions (DARs). **(A)** Distance to the closest transcription start site (TSS) of all accessible regions and newly open and closed regions. **(B)** Heatmap representation of normalized ATAC-seq signal in parental and doxorubicin-resistant MCF7 cells over DARs. The top panel shows read signal over the 11,686 hyper-accessible regions in MCF7-DR cells, while the bottom panel shows read signal over the 6,542 hypo-accessible regions. Signals within 1.5 Kb surrounding the center of DARs are displayed in descending order. **(C)** Profiles of normalized tag density across a genomic window of ±1.5 Kb surrounding the center of hyper- (top) and hypo-accessible (bottom) regions. **(D)** Pie chart showing the proportion of hyper- (top) and hypo-accessible (bottom) sites within the indicated genomic regions: introns, exon, intergenic regions, 3′ UTR, 5′ UTR, promoters-TSS, TES and no-coding regions. Peak summits located up to 1 Kb upstream and 100 bp downstream of the TSS are determined as promoter-TSS region. **(E)** Venn diagram illustrating the overlap among DEGs and the nearest genes of differentially accessible regions. **(F)** Box plots for mRNA expression levels of DEGs relative to hyper- (left) and hypo-accessible (right) regions. Notches of the boxes indicate medians. The *P* values were calculated by Wilcoxon’s signed-rank test. ****P < 0.0001. **(G)** Correlation analysis between DARs and their nearest DEGs. Each blue-colored dot represents a gene that is significantly differentially expressed and associated with chromatin accessibility changes. The top- and bottom-ranked 10 DEGs are labeled and shown in different colors based on the log_2_(FC) of the average FPKM. The Pearson’s correlation coefficient (r) and the corresponding *P* value are shown.

In order to determine whether the alteration of chromatin accessibility was consistent with transcription changes, we assessed differential accessibility between the parental and doxorubicin-resistant MCF7 breast cancer cells (MCF7-DR) across the genome. In total, 11,686 accessible regions were > 1.5-fold more accessible in MCF7-DR cells (Figures 2D, 3A–C). Conversely, 6,542 regions were > 1.5-fold more accessible in the MCF7 cells, and thus we defined these chromatin states as “hyper-accessible” and “hypo-accessible.” In general, these DARs were largely intragenic and gene distal (Figures 3A,D and Supplementary Table 4), with relatively few promoter-proximal regions exhibiting differential accessibility, suggesting that DARs might represent distal regulatory elements such as enhancers, which were often located far away from the genes they control. Moreover, it is worth noting that there were some wide continuous areas with decreased chromatin accessibility on chr7 and chr13 (Figure 2D), and most of the DEGs located in these regions were significantly down-regulated (Supplementary Table 2), suggesting that changes in chromatin accessibility are closely associated with the concomitant differential gene expression.

### The DARs Were Positively Correlated With Nearest DEGs

To acquire the potential links between DARs and DEGs, we assigned differentially accessible sites to the nearest genes according to their locations in the hg19 genome. Combining RNA-seq data, a total of 1,062 and 785 nearby DEGs were identified to be associated with the hyper- and hypo-accessible regions, respectively (Figure 3E). Interestingly, our results showed that most of the DEGs associated with hyper-accessible regions were significantly up-regulated, while the DEGs associated with hypo-accessible regions displayed significantly down-regulated RNA levels in all biological replicates (Figure 3F), indicating that genes within the open chromatin regions tended to have higher expression levels. Upon further analysis, it was confirmed that the changes in gene expression were positively associated with DARs located within 100 Kb of the TSS (Pearson correlation coefficient *r* = 0.6), suggesting that the alterations in chromatin accessibility contribute to differential expression of the genes associated with doxorubicin in MCF7-DR cells, such as *MT1E*, *GPX1*, *LDHB* and *TPM2* (Figure 3G).

### Key Signaling Pathway Associated DEGs That Positively Correlated With DARs

**FIGURE 4:**
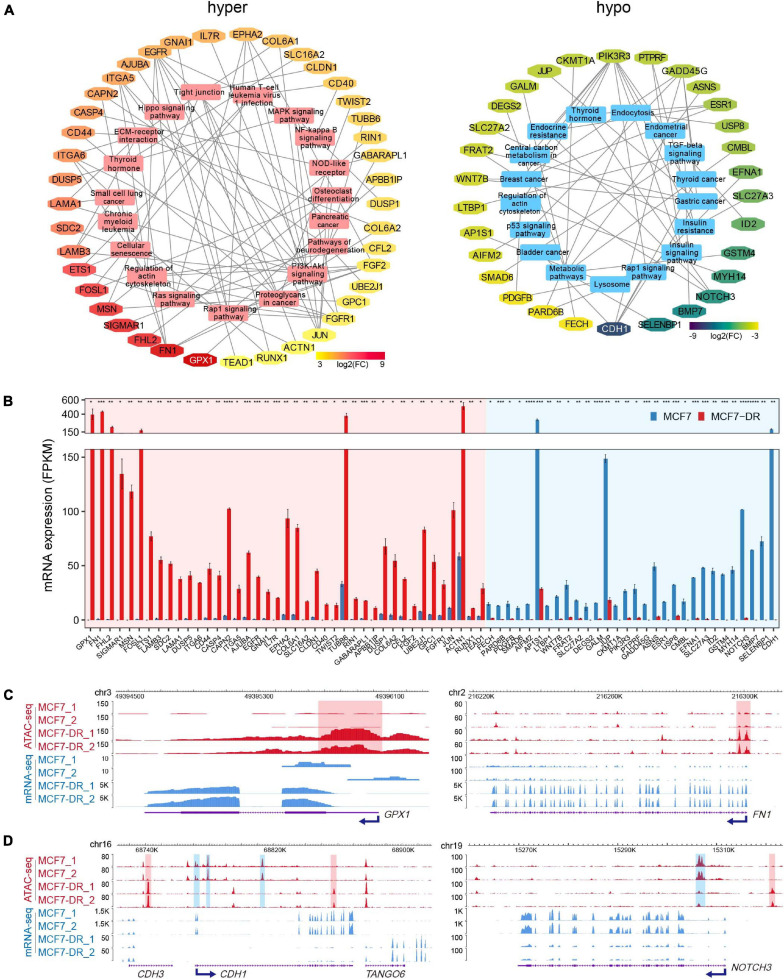
Key KEGG pathways associated DEGs related to DARs. **(A)** Top 20 KEGG pathways associated DEGs related to hyper- (left) and hypo-accessible (right) regions. DEGs are shown in different colors according to log_2_(FC). **(B)** Bar plot showing average expression levels (FPKM) of DEGs related to hyper- (red shaded area) and hypo-accessible (blue shaded area) regions which enriched in top 20 KEGG pathways. DEGs are listed in a descending order based on log_2_(FC). The *P* values were calculated using Student’s *t* test in R. *P < 0.05, **P < 0.01, ***P < 0.001, and ****P < 0.0001. **(C)** The WashU Epigenome Browser tracks show ATAC-seq (red) and mRNA-seq (blue) signals of representative up-regulated *GPX1* and *FN1* genes. The hyper-accessible regions are shaded with red. **(D)** Genomic snapshots of ATAC-seq (red) and mRNA-seq (blue) signal of representative down-regulated *CDH1* and *NOTCH3* genes. The hypo-accessible regions are shaded with blue.

To identify potential key DEGs and signal pathways associated with DARs in MCF7-DR cells, we performed KEGG pathway enrichment analysis for DEGs associated with hyper- and hypo-accessible regions respectively. It was revealed that the hyper-accessible regions associated up-regulated DEGs were significantly enriched in small cell lung cancer, cell cycle, MAPK and Rap1 signaling pathways, while the hypo-accessible regions associated down-regulated DEGs mainly involved in endocrine resistance, lysosome, breast cancer, TGF-beta and p53 signaling pathways (Supplementary Figures 2, 4A). By integrating ATAC-seq and RNA-seq data, we obtained a total of 71 key DEGs enriched in the top 20 KEGG pathways with an absolute value of log_2_(FC) > 3 (Figures 4A,B), including 41 up-regulated and 30 down-regulated DEGs positively associated with hyper- and hypo-accessible regions respectively, such as *GPX1*, *FN1*, *FOSL1*, *CDH1*, *NOTCH3, WNT7B*, and *MT1E* (Figures 4C,D and Supplementary Figure 3). This study indicated that global changes in chromatin accessibility affected the gene expression of many components of signaling cascades, and played a critical role in acquired resistance to doxorubicin in MCF7 breast cancer cells.

### TF Families Related to Doxorubicin-Induced Chromatin Accessibility Changes

**FIGURE 5:**
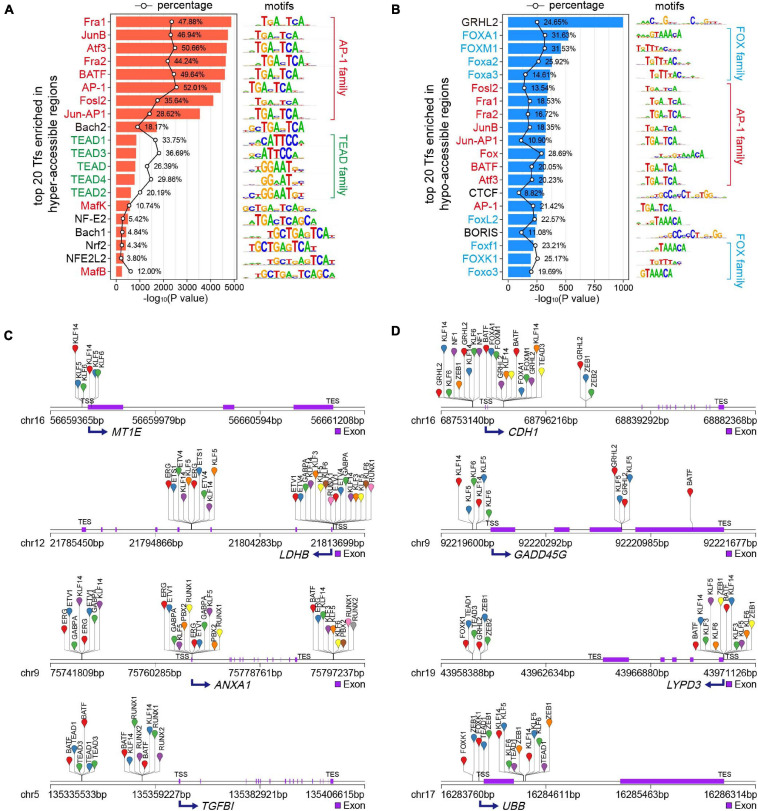
Transcription factor (TF) motif enrichment analysis of DARs. **(A,B)** Top 20 enriched known TF motifs of hyper- **(A)** and hypo-accessible **(B)** sites, with *P* values estimated from HOMER v4.10. The AP-1, TEAD and FOX family members were colored red, green and blue, respectively. The polygonal chain in black shows the percentages of target sequences with TF motifs. **(C,D)** Putative TF occupancy of representative up- **(C)** and down-regulated **(D)** DEGs positively correlated to chromatin accessibility changes.

TFs are molecules involved in regulating gene expression through binding to *cis*-regulatory specific sequences, known as motifs, in the promoters or enhancers of their target genes (Tsankov et al., 2015; Lambert et al., 2018). In most cases, chromatin accessibility to DNA is a prerequisite for TF binding (Huertas et al., 2020), which plays an important role in development and diseases (Whyte et al., 2013; Denny et al., 2016; Lambert et al., 2018; Tome-Garcia et al., 2018; Wang et al., 2018). To examine potential TFs located in the DARs in MCF7-DR cells, we performed TF enrichment analysis using motif discovery software HOMER (v4.10) (Heinz et al., 2010). As TFs binding to their cognate motifs, often obligates nucleosome eviction and creation of accessible chromatin regions, the integration of known TF motifs and chromatin accessibility data from ATAC-seq can predict TF occupancy on the chromatin across the whole genome (Buenrostro et al., 2013; Bao et al., 2015; Qu et al., 2017; Corces et al., 2018). The ± 200 bp flanking sequences around ATAC-seq peak summits in DARs were scanned for TF motif occurrences and TF binding sites (TFBSs). By default, 268 and 266 potential TFs were identified (*P* < 0.01) in hyper- and hypo-accessible regions, respectively (Supplementary Table 5). The activator protein-1 (AP-1) family members, including Jun (c-Jun, JunB, JunD), Fos (c-Fos, FosB, FosB2, Fra-1, and Fra-2), ATF (ATFa, ATF2, ATF3, BATF), and Maf (Maf1, MafA, MafK) proteins, were significantly enriched in both hyper- and hypo-accessible regions (Figures 5A,B), which always bind to the DNA sequence TGACTCA and play a critical role in various cellular events including proliferation, differentiation and apoptosis (Eferl and Wagner, 2003; Papoudou-Bai et al., 2017; Lambert et al., 2018). In addition, it was revealed that the hyper-accessible regions were also enriched with transcriptional enhanced associate domain (TEAD) family TFs, while the hypo-accessible regions were enriched for forkhead box (FOX) family members (Figures 5A,B and Supplementary Table 5).

To obtain potential regulators of DEGs associated with doxorubicin resistance in breast cancer cells, we performed the analysis of the binding sites of top 50 TFs (Supplementary Table 5) enriched in DARs using HOMER (v4.10) (Heinz et al., 2010). The data results showed that the hyper-accessible sites relative to TSS of key up-regulated DEGs, including *MT1E*, *LDHB*, *ANXA1*, and *TGFBl*, were significantly enriched by TFBSs of KLF, ETV, RUNX and TEAD family members (Figure 5C). Meanwhile, the FOX family members and GRHL2 were mainly bound to the hypo-accessible sites associated with down-regulated DEGs, such as *CDH1*, *GADD45G*, *LYPD3*, and *UBB* (Figure 5D).

### Differentially Expressed Transcription Factors (DETFs) and Their Target DEGs Related to DARs

**FIGURE 6:**
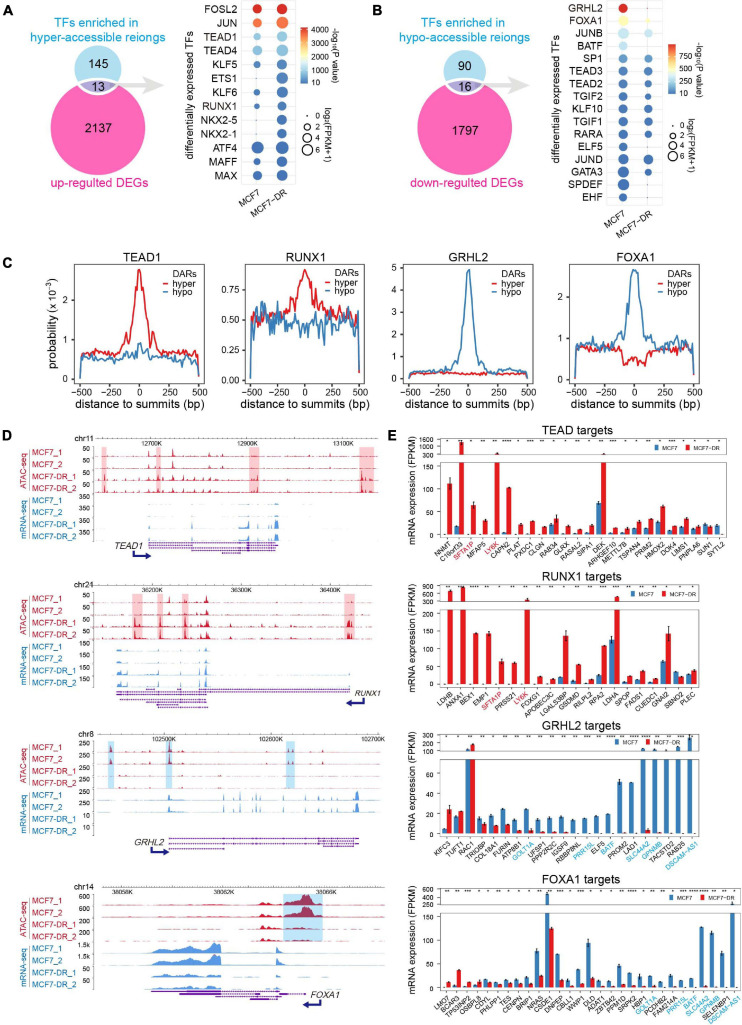
Differentially expressed TFs (DETFs) and their target DEGs. **(A)** Venn diagram (left) showing overlap of the corresponding TFs for each motif in hyper-accessible regions and up-regulated DEGs. The dot plot (right) showing the identified up-regulated TFs. The color of each dot represents the *P* value of enrichment (calculated using the cumulative binomial distribution in HOMER v4.10) for each motif and cell type, and the size of each dot represents the gene expression (FPKM) of the enriched motif cognate TFs. Only TFs motif enrichment *P* value < 10^– 10^ were included. **(B)** Venn diagram (left) showing overlap of the corresponding TFs for each motif in hypo-accessible regions and down-regulated DEGs. The dot plot (right) showing the identified down-regulated TFs. **(C)** Distribution probability of TEAD1, RUNX1, GRHL2 and FOXA1 binding motifs around ATAC-seq peak summits within DARs. **(D)** The WashU Epigenome Browser views show the hyper-accessible chromatin regions (red shaded area) near two up-regulated TFs (*TEAD1* and *RUNX1*), and the hypo-accessible chromatin regions (blue shaded area) near two down-regulated TFs (*GRHL2* and *FOXA1*). **(E)** Bar charts showing the expression levels of potential TF (TEAD1, RUNX1, GRHL2 and FOXA1) target DEGs in DARs predicted using HOMER. Only DEGs with TFBSs in the promoter-TSS region were included. Genes are listed in a descending order based on log_2_(FC). TEAD1 and RUNX1 common targets were colored in red, while GRHL2 and FOXA1 common targets were colored in blue. The *P* values were calculated using Student’s *t* test in R. *P < 0.05, **P < 0.01, ***P < 0.001, and ****P < 0.0001.

In order to identify the DETFs that might be involved in the regulation of chromatin accessibility and gene transcription, the enriched motif cognate TFs (*P* < 10^–10^) were intersected with the DEGs (Figures 6A,B). A total of 13 up-regulated TFs enriched in the hyper-accessible regions and 16 down-regulated TFs enriched in the hypo-accessible regions. For instance, besides AP-1 family members, TEAD1 and RUNX1 were significantly up-regulated and enriched in hyper-accessible regions, while down-regulation of GRHL2 and FOXA1 might be responsible for global decreases in chromatin accessibility. As expected, further analysis demonstrated that TEAD1 and RUNX1 showed higher binding probability around peak summits in hyper-accessible regions, while GRHL2 and FOXA1 were generally distributed more frequently in hypo-accessible regions (Figure 6C). Moreover, the differential expression patterns of these TFs were positively correlated with the alterations in chromatin accessibility (Figure 6D). The majority of predicted target genes of TEAD1 and RUNX1 were significantly up-regulated, including *SFTA1P* and *LY6K*, while most of GRHL2 and FOXA1 targets were down-regulated, such as *SLC44A2* and *GPNMB* (Figure 6E).

### Loss of the Pioneer Factor FOXA1 Expression Might Be Responsible for Doxorubicin Resistance in Breast Cancer

**FIGURE 7:**
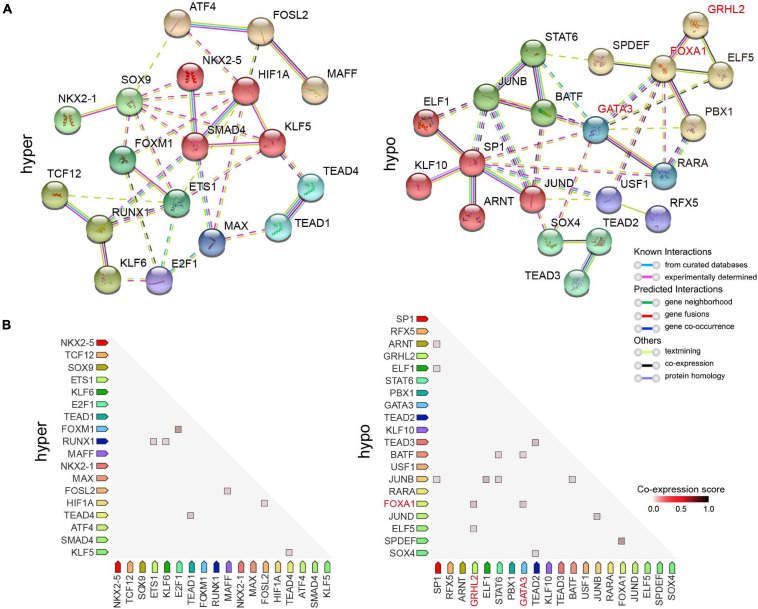
Protein-protein interaction (PPI) and co-expression network analysis of DETFs. **(A)** PPI network showing DETFs identified by HOMER enriched in hyper- (left) and hypo-accessible (right) regions. Network nodes indicate proteins, and the colored edges represent evidence of different types of protein-protein interactions. Strong relationships and networks were clustered using Markov cluster algorithm by default inflation parameters and indicated by solid lines. **(B)** Observed co-expression in Homo sapiens identified by STRING for DETFs associated with DARs.

It has been recently reported that FOXA1/GRHL2 collaboration could establish a targetable pathway in endocrine therapy-resistant breast cancer (Cocce et al., 2019), and facilitate depositing H3K4me1 at potential enhancer elements by recruiting the chromatin modifier MLL3 (Jozwik et al., 2016). *GATA3* and *FOXA1* are marker genes that define an ESR1-positive breast cancer (Cancer Genome Atlas, 2012). GATA3 acts upstream of FOXA1, loss of which results in changes in FOXA1 binding and alteration of gene expression (Theodorou et al., 2013). Our data demonstrated that a large proportion of the predicted target DEGs were potentially co-regulated by both FOXA1 and GRHL2 in MCF7 cells (Figure 6E), such as *SLC44A2*, *GPNMB*, *BATF*, and *GOLT1A*, which suggested that loss of FOXA1/GRHL2 collaboration might be responsible for the down-regulation of critical genes in doxorubicin-resistant breast cancer cells. To identify DETFs (*P* < 10^–2^) that might act coordinately to control their gene expression, we constructed a protein-protein interaction (PPI) network using the STRING database (Szklarczyk et al., 2017), which serves as an entry point for a better interpretation of relationships between different TFs on a genome-wide scale. It revealed that clustered SMAD4/KLF5/HIF1A and FOXA1/GRHL2/ELF5 potentially worked together to regulate gene expression in hyper- and hypo-accessible regions, respectively (Figure 7A). Moreover, there were higher observed co-expression scores for FOXM1/E2F1, FOXA1/GRHL2, FOXA1/GATA3 (Figure 7B), suggesting that absence of FOXA1 might contribute to doxorubicin resistance in breast cancer cells through collaboration with the down-regulated *GRHL2* and/or *GATA3*.

**FIGURE 8:**
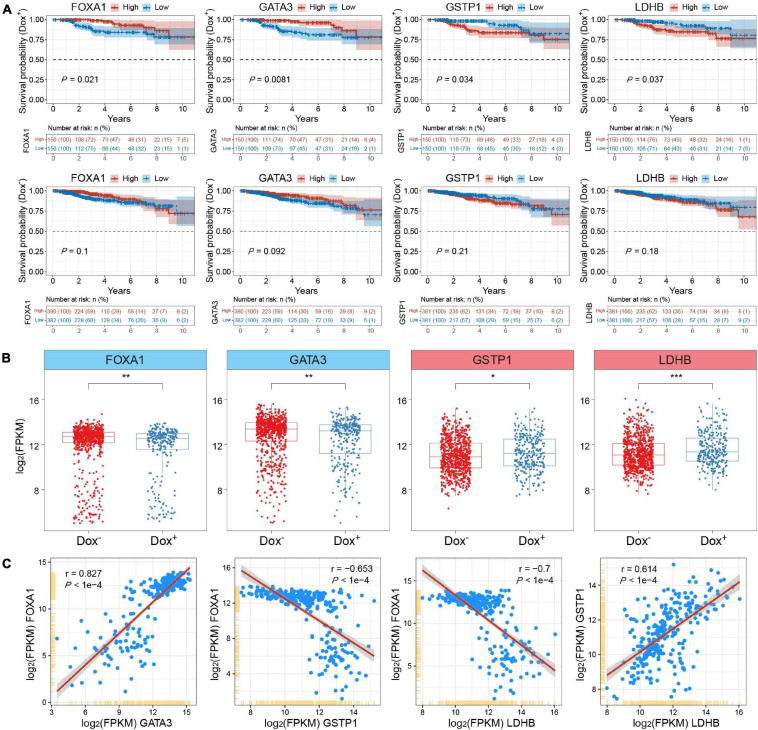
Integrative analysis of clinical and genomic data from TCGA-BRCA datasets. **(A)** Kaplan–Meier survival analyses for *FOXA1*, *GATA3*, *GSTP1* and *LDHB* of breast invasive carcinoma (BRCA) patients treated with (Dox^+^; top panel) and without (Dox^–^; bottom panel) doxorubicin (adriamycin). Expression values were sorted according to high and low expression (FPKM) from the median of the corresponding genes. The shaded area indicates the 95% confidence interval. All the survival duration was censored to 10 years. Statistical analysis was performed by the log-rank test, and *P* < 0.05 was statistically significant. **(B)** Box plots showing the mRNA expression levels (FPKM) of *FOXA1*, *GATA3*, *GSTP1* and *LDHB* in Dox^+^ and Dox^–^ breast cancer subgroups derived from TCGA datasets. The *P* values were calculated by Wilcoxon’s signed-rank test. *P < 0.05, **P < 0.01, and ***P < 0.001. **(C)** Correlation analysis of mRNA expression levels among *FOXA1*, *GATA3*, *GSTP1* and *LDHB* in the Dox^+^ breast cancer subgroup. The Pearson’s correlation coefficients (r) and *P* values were calculated using R packages. The shaded gray area around the red line represents the 95% confidence interval. The *P* value less than 0.05 was considered to be statistically significant.

To further testify the expression patterns and the clinical significance of critical DEGs related to chromatin accessibility changes in MCF7-DR cells as well as predict their role in the development of acquired resistance to doxorubicin in breast cancer, we retrieved the genomic and clinical data from the Cancer Genome Atlas Breast Invasive Carcinoma (TCGA-BRCA) and analyzed expression levels of the DEGs and their impact on the prognosis of breast cancer patients treated with (Dox^+^; *n* = 300) and without (Dox^–^; *n* = 762) doxorubicin alone. Remarkably, the results showed that lower expression of several significantly down-regulated genes in MCF7-DR cells was verified to be significantly (*P* < 0.05) associated with the poor prognosis of the Dox^+^ patients when compared to the Dox^–^ patients, including *FOXA1*, *GATA3*, *RARA*, and *SLC27A*2 (Figure 8A and Supplementary Figure 4). On the contrary, higher expression of up-regulated *GSTP1* and *LDHB* was related with the poor prognosis of Dox^+^ patients (Figure 8A). The expression levels of *FOXA1* and *GATA3* were significantly (*P* < 0.05) lower in the Dox^+^ patients than Dox^–^, while *GSTP1* and *LDHB* expression levels were higher in the Dox^+^ patients (Figure 8B). Similarly, the expression patterns of *RARA* and *SLC27A*2 were generally consistent with the previously described transcriptomic changes in MCF7-DR cells (Figure 1B, Supplementary Figure 5, and Supplementary Table 2). Moreover, in the Dox^+^ patients, there was a strong positive correlation between the mRNA expression levels of *FOXA1* and *GATA3* (*r* = 0.827), *GSTP1* and *LDHB* (*r* = 0.614), *FOXA1* and *SPDEF* (*r* = 0.857), while *FOXA1* expression was negatively correlated with *GSTP1* (*r* = −0.653) and *LDHB* (*r* = −0.7) as expected (Figure 8C and Supplementary Figure 6). These results revealed that some of the above-mentioned DEGs associated with chromatin accessibility changes, such as *FOXA1*, *GATA3* and *GSTP1*, played a critical role in the development of doxorubicin resistance in breast cancer cells, and might be important indicators for judging the prognosis or a therapeutic target in breast cancer patients treated with doxorubicin.

## Discussion

Despite a few molecular mechanisms involved in the process of doxorubicin resistance in cancer have been reported (Huang et al., 2018; Lovitt et al., 2018; Stefanski et al., 2019; Jin et al., 2020; Wang et al., 2020; Mirzaei et al., 2021; Yang et al., 2021), drug resistance is almost inevitable in advanced breast cancer patients, which remains a major unresolved issue in the treatment of cancer patients. An increasing number of studies have revealed that the alteration in chromatin accessibility plays a critical role in acquired resistance against cancer drugs (Jing et al., 2018; Zhang et al., 2020), but we still lack precise knowledge of how accessible chromatin state define transcriptional regulatory networks and which are the regulatory elements involved in doxorubicin resistance in breast cancer cells.

In the present study, we integrated chromatin accessibility and transcription analysis in parental and doxorubicin-resistant MCF7 cells *via* ATAC-Seq in combination with RNA-seq, which are mutually authenticating. Compared to existing FAIRE-seq, DNase-seq and MNase-seq technologies, ATAC-seq offers substantial advantages and becomes the most widely used method to analyze the genome-wide profile of chromatin accessibility due to its low input cell number requirement, simplicity, speediness and fine accuracy (Buenrostro et al., 2013).

Based on the analysis of differential expression level of mRNA, we identified several dramatically up-regulated genes associated with doxorubicin resistance, such as glutathione S-transferases P1(*GSTP1*), metallothionein gene family (*MT1E*, *MT1M*, and *MT1L*) and glutathione peroxidase 1 (*GPX1*). It has been reported that overexpression of metallothionein confers resistance to anti-cancer drugs (Kelley et al., 1988); *GPX1* promotes cisplatin resistance *via* ROS-Induced activation of PI3K/AKT pathway in non-small cell lung cancer (Chen et al., 2019). Up-regulation of GSTP1 maintains resistance to doxorubicin through inhibition of PI3K/AKT/mTOR activity to promote autophagy in breast cancer cells (Dong et al., 2019); Simultaneously, there were a lot of significantly down-regulated genes in MCF7-DR cells, including trefoil factor 1 (*TFF1*), ubiquitin B (*UBB*), and SAM pointed domain containing ETS TF (*SPDEF*). Furthermore, from a perspective of epigenetic regulation, we analyzed the changes in the expression of known histone-modifying enzymes especially. The results suggested that the up-regulation of histone deacetylase 2 (*HDAC2)* and histone methyltransferase *EZH2* was potentially involved in the down-regulation of certain doxorubicin-resistant genes.

In general, our RNA-seq results are broadly consistent with previous studies regarding the identification of DEGs and pathway enrichment analysis. However, as far as we know, most of the identified DEGs have not yet been studied in sufficient detail to identify their genetic functions involved in doxorubicin-resistant breast cancer.

Genome-wide analysis of chromatin accessibility revealed that ATAC-Seq signal was significantly enriched in promoter-TSS and distal regulatory elements such as enhancers. Combined with RNA-seq data, a significant positive correlation (*r* = 0.6) was found between DARs and DEGs, validating our expectation that increased chromatin accessibility results in higher expression of critical genes associated with doxorubicin resistance, such as *MT1E*, *GPX1*, and *TPM2*. In the same way, condensed chromatin, known as closed chromatin, restricts the binding of TFs and transcriptional regulators to the promoter-TSS and/or enhancer, which leads to gene silencing, including *TFF1*, *UBB* and *CDH1*. Furthermore, we screened the DEGs that were significantly positively correlated with chromatin accessibility changes, and then, Cytoscape (version 3.7.1) software was used to display their enriched functional pathways.

Transcription factors play an important role in cancer drug resistance (Fan et al., 2017; Lambert et al., 2018; Huh et al., 2019; Bi et al., 2020). By analyzing the accessibility of chromatin, we cannot only identify the regulatory regions but also infer TF motifs. Therefore, we performed known motif analysis of the DARs to identify potential TFs and their target DEGs using the motif discovery software HOMER. We found that, besides the AP-1 family, members of TEAD and FOX families were significantly enriched in hyper- and hypo-accessible regions in MCF7-DR cells. The AP-1 is another family of bZIP TFs and comprises multiple proteins that bind as heterodimers (Lambert et al., 2018), which is also a common TF family in motif analysis of ATAC-Seq data. In this study, we would like to pay more attention to other TFs with important regulatory functions. Finally, we identified several significantly up- and down-regulated TFs (*P* < 10^–10^) associated with the hyper- and hypo-accessible regions, respectively, such as TEAD1, RUNX1, GRHL2 and FOXA1. It has been previously reported that the pioneers TFs FOXA1 and GATA3 mediated ESR1 binding by shaping enhancer accessibility (Theodorou et al., 2013); FOXA1 collaborates with GRHL2 and MLL3 to establish a targetable collateral pathway in endocrine therapy-resistant breast cancer (Werner et al., 2013; Cocce et al., 2019). The PPI network analysis showed that GATA3/FOXA1/GRHL2 exhibited strong correlations, suggesting loss of *GATA3*/*FOXA1*/*GRHL2* expression might play an important role in the down-regulation of key genes in MCF7-DR cells, such as *SLC44A2*, *BATF*, and *PRR15L*. Moreover, the analysis of genomic and clinical data from TCGA-BRCA further revealed that low expression levels of *FOXA1* and *GATA3* in patients treated with doxorubicin alone experienced significantly worse clinical outcomes, while *GSTP1* and *LDHB* had opposite prognostic effects. As was expected, all of them exhibited similar gene expression patterns in both DOX^+^ breast cancer patients and MCF7-DR cells.

Most of the previous research on drug resistance has focused on the up-regulated genes but paid relatively little attention to the down-regulated genes. Our findings suggest that down-regulation or loss of certain regulators may also play an important role in promoting the development of cancer drug resistance. According to previous studies and our data analysis, we concluded that chromatin accessibility changes could promote or inhibit the expression of critical genes associated with doxorubicin resistance by affecting the DNA-binding ability of TFs and transcriptional regulators in promoter and/or enhancer. It is probably that, in doxorubicin-resistant breast cancer cells, loss of the pioneer TF FOXA1 leads to a significant decrease in chromatin openness, which impairs the ability of co-regulators GATA3 and/or GRHL2 to occupy their specific binding sites in the promoter and/or enhancer region of specific genes, such as *BATF*, *SLC44A2*, and *GPNMB*. Besides, the opposite changes in gene expression of *FOXA1* and *GSTP1* might be potential therapeutic targets for overcoming or reversing doxorubicin resistance in breast cancer.

## Conclusion

The genome-wide profiling of open chromatin by ATAC-seq represents a novel strategy to study accessibility dynamics and transcriptional regulation in cancer drug resistance. The application of this technique allows us to identify the accessible regulatory elements and TFs that are probably responsible for the acquired resistance to doxorubicin in breast cancer cells. Our ATAC-seq data contribute to close the loop for the association between TF binding, transcriptional and chromatin states, providing new insights into non-genetic mechanisms of doxorubicin resistance and putative targets for epigenetic therapy.

## Data Availability Statement

All relevant datasets supporting the key findings of this study are available within the article and its Supplementary Material or from the corresponding author on reasonable request. All sequencing data have been deposited in the GEO repository: GSE174152.

## Author Contributions

GW and XW conceived the idea. XW prepared samples and generated the data. XW analyzed the data with assistance from GW and JY. XW wrote the manuscript. GW and BS supervised the study and revised the manuscript. JY provided helpful comments on this study. All authors reviewed and approved the final manuscript.

## Conflict of Interest

The authors declare that the research was conducted in the absence of any commercial or financial relationships that could be construed as a potential conflict of interest.

## Publisher’s Note

All claims expressed in this article are solely those of the authors and do not necessarily represent those of their affiliated organizations, or those of the publisher, the editors and the reviewers. Any product that may be evaluated in this article, or claim that may be made by its manufacturer, is not guaranteed or endorsed by the publisher.

## Footnotes

1
http://www.bioinformatics.babraham.ac.uk/projects/fastqc/

2
https://broadinstitute.github.io/picard/

3
http://www.cytoscape.org/

4
https://www.R-project.org/

5
https://epgg-test.wustl.edu/browser/
