# Supplementary material for: Integrated Chromatin Accessibility and Transcriptome Landscapes of Doxorubicin-Resistant Breast Cancer Cells
Source: Front Cell Dev Biol. 2021 Jul 30;9:708066. doi: 10.3389/fcell.2021.708066 (PMC8363264; doi:10.3389/fcell.2021.708066)
Supplement: Supplementary file 1 [file Data_Sheet_1.docx]

Supplementary Material

## Supplementary Figures


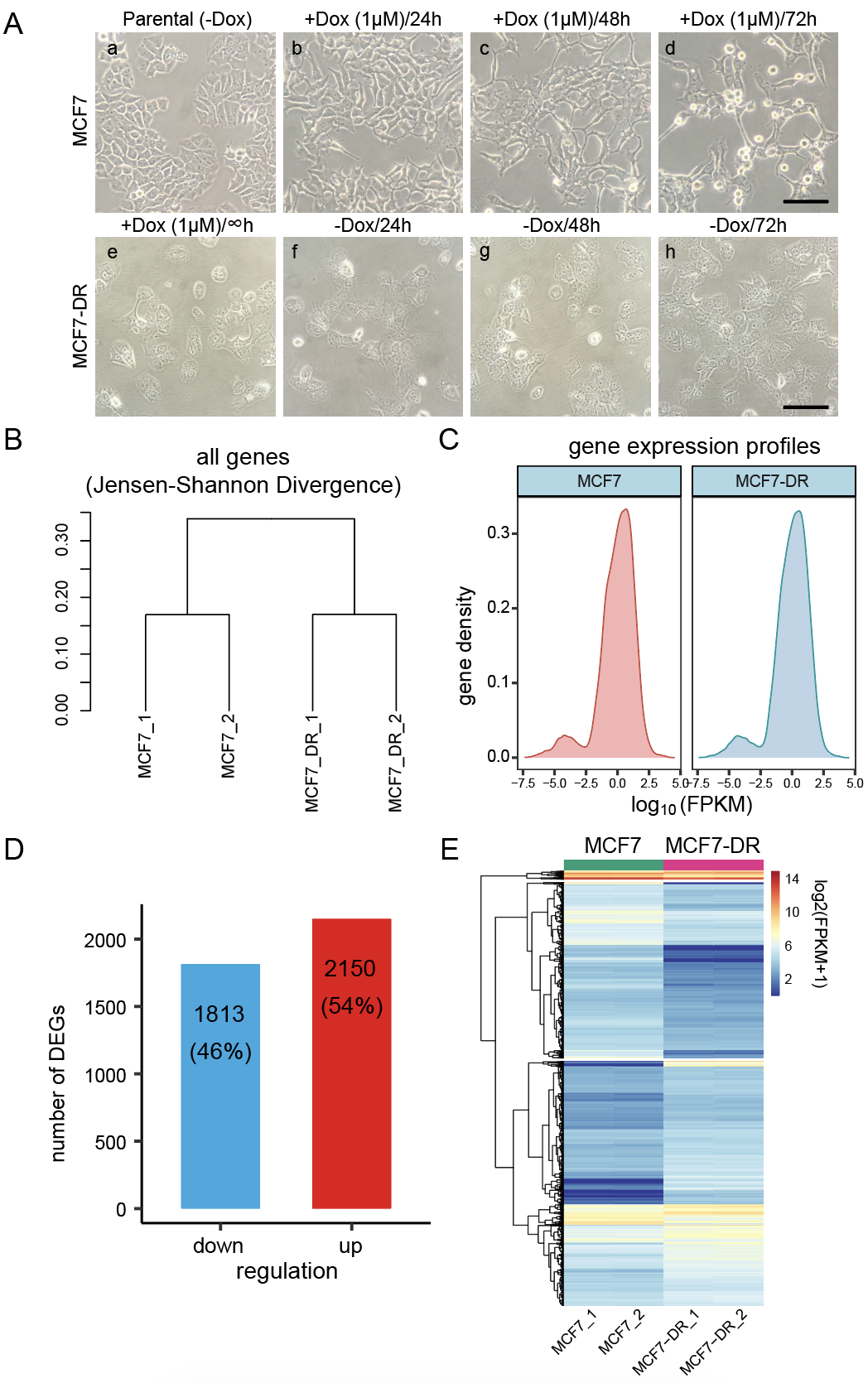


**Supplementary Figure S1.** **Gene expression profiling analysis of the parental and doxorubicin-resistant MCF7 breast cancer cells.**

(A) (a) showing the morphology of parental MCF7 cells cultured in the medium without doxorubicin (-Dox); (b-d) indicate the MCF7 cells treated with 1μM doxorubicin (+Dox) for 24h, 48h and 72h; (e) indicates the morphology of doxorubicin-resistant MCF7 cells (MCF7-DR) cultured in medium with 1μM doxorubicin (+Dox) for more than several months (∞h); (f-h) showing the MCF7-DR cells cultured in the medium without doxorubicin (-Dox) for 24h, 48h and 72h. The MCF7 cells under condition (a) and the MCF7-DR cells under condition (h) were used as the control group and experimental group, respectively, for any further experiments in this study. Scale bar, 50 µm.

(B) The expression levels (FPKM values) across all gene protein coding transcripts (hg19 reference genome) were used to calculate Jensen-Shannon Divergence (JSD).

(C) The average gene expression profiles of two biological replicates in each cell line.

(D) Bar plot showing the number and percentage of significantly up- and down-regulated genes in doxorubicin-resistant MCF7 (MCF7-DR) compared with parental cells.

(E) Heatmap showing the gene expression levels of 3963 genes differentially expressed between MCF7 and MCF7-DR cells. The differentially expressed genes (DEGs) filtered by q value < 0.05, fold change (FC) > 1.5 and average FPKM > 10 at least one group.


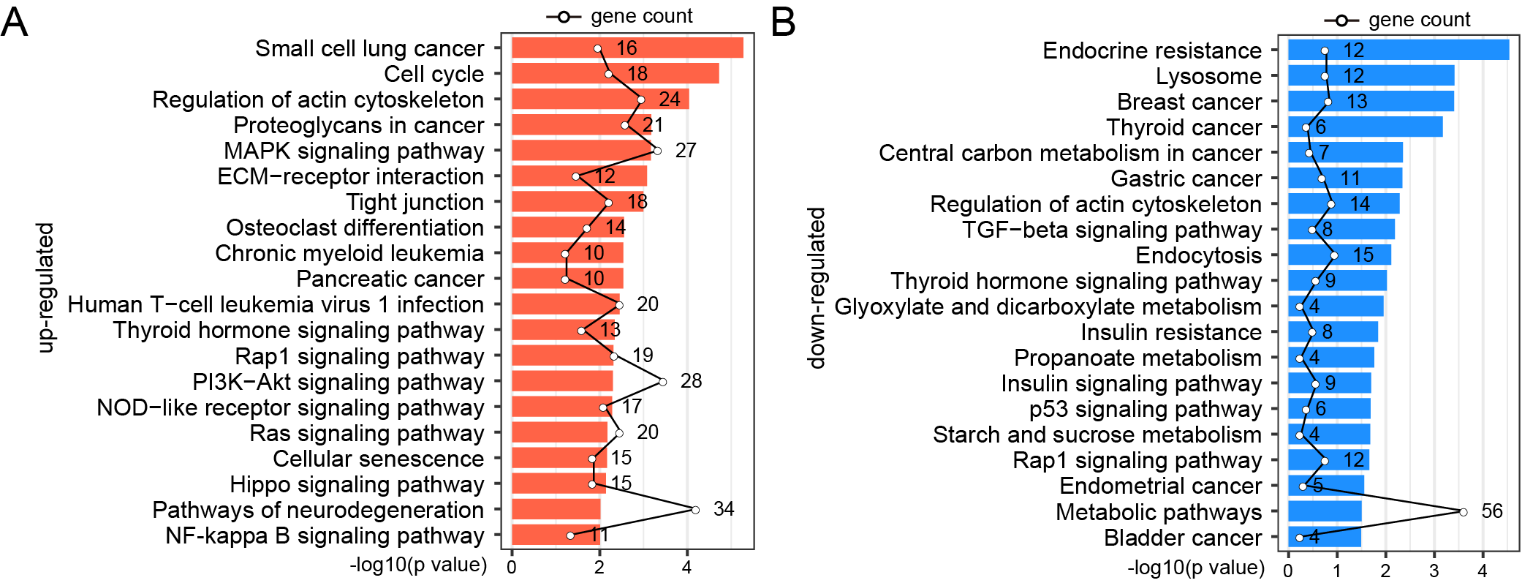


**Supplementary Figure S2.** **KEGG pathway analysis of DEGs related to differentially accessible regions (DARs).**

(A and B) Top 20 KEGG pathway associated DEGs related to hyper-accessible (A) and hypo-accessible (B) regions. The polygonal chain in black shows the count of DEGs enriched in each KEGG pathway.


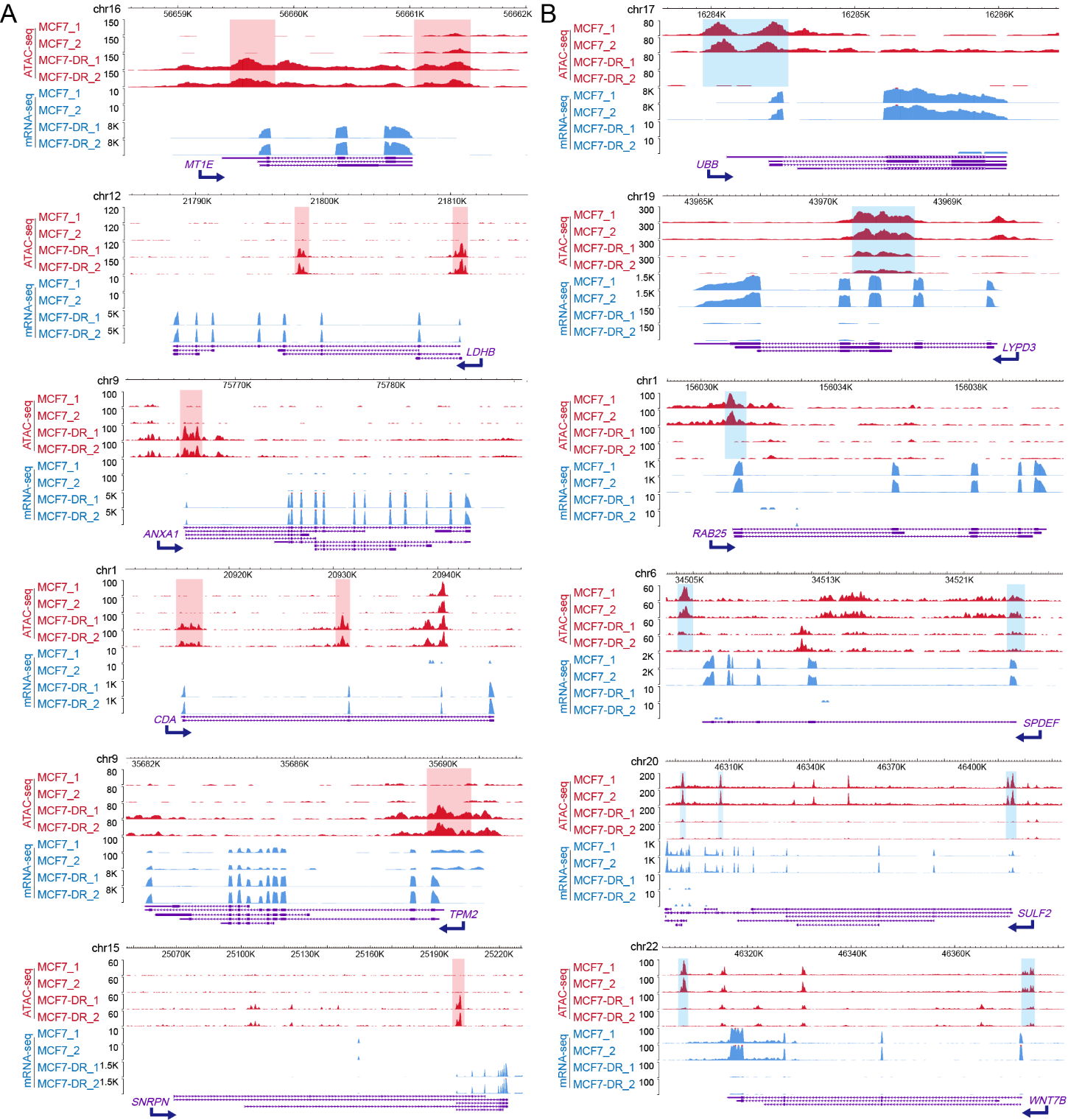


**Supplementary Figure S3.** **DEGs associated with key signal pathway and positively correlated to DARs.**

(A and B) The WashU Epigenome Browser views show ATAC-seq (red) and mRNA-seq (blue) signal of representative up-regulated (A) and down-regulated (B) DEGs associated with top 20 KEGG signal pathways and related to hyper- (red shade area) and hypo-accessible (blue shade area) regions, respectively.


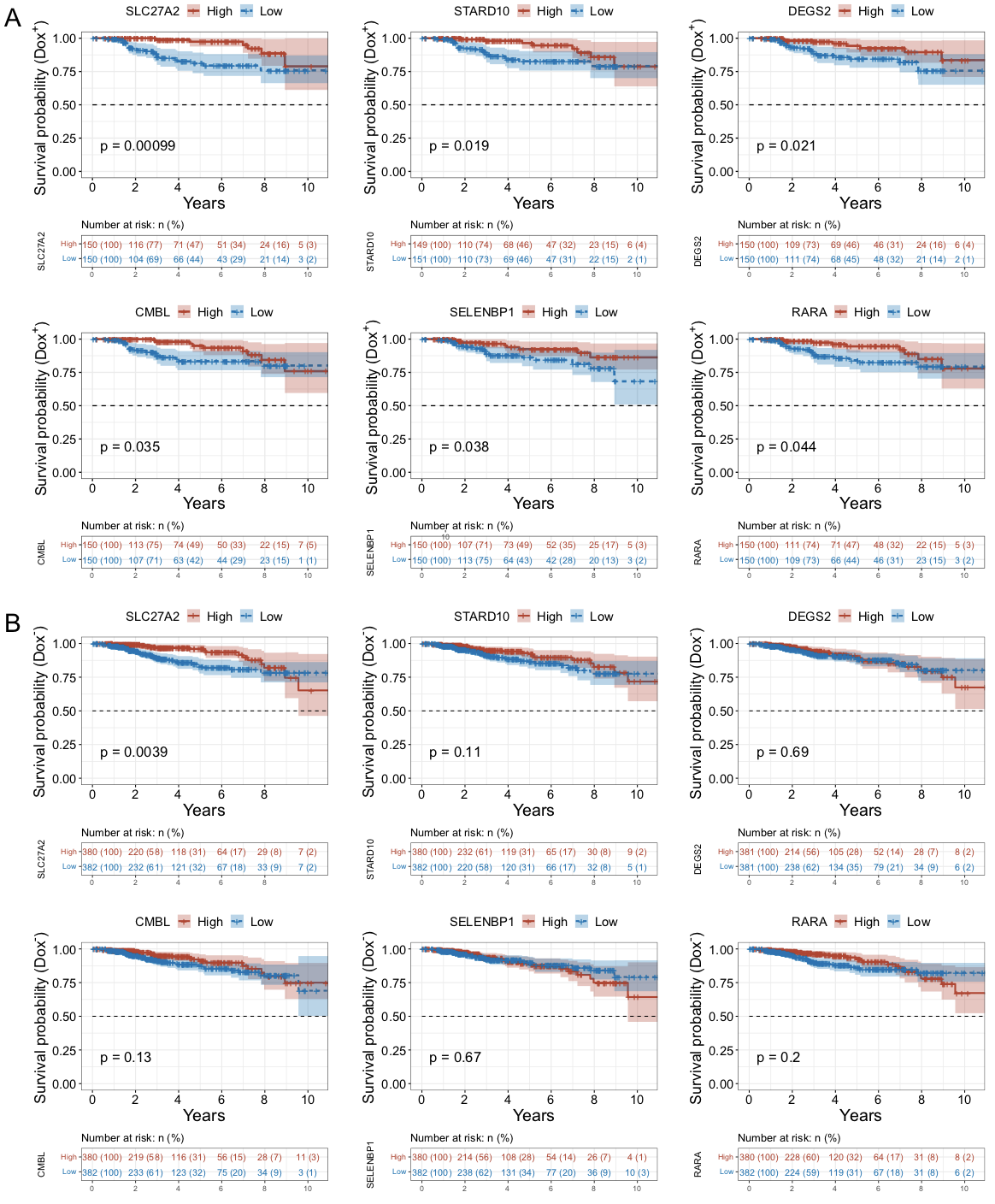


**Supplementary Figure S4. Overall survival analysis of breast cancer patients from TCGA datasets.**

(A and B) Kaplan-Meier survival analyses for significantly down-regulated SLC27A2, STARD10, DEGS2, CMBL, SELENBP1 and RARA of breast invasive carcinoma (BRCA) patients treated with (Dox+; n = 300; A) and without (Dox-; n = 762; B) doxorubicin (adriamycin). Expression values were sorted according to high and low expression (FPKM) from the median of the corresponding genes. The shaded area indicates the 95% confidence interval. All the survival duration was censored to 10 years. Statistical analysis was performed by the log-rank test, and P < 0.05 was statistically significant.


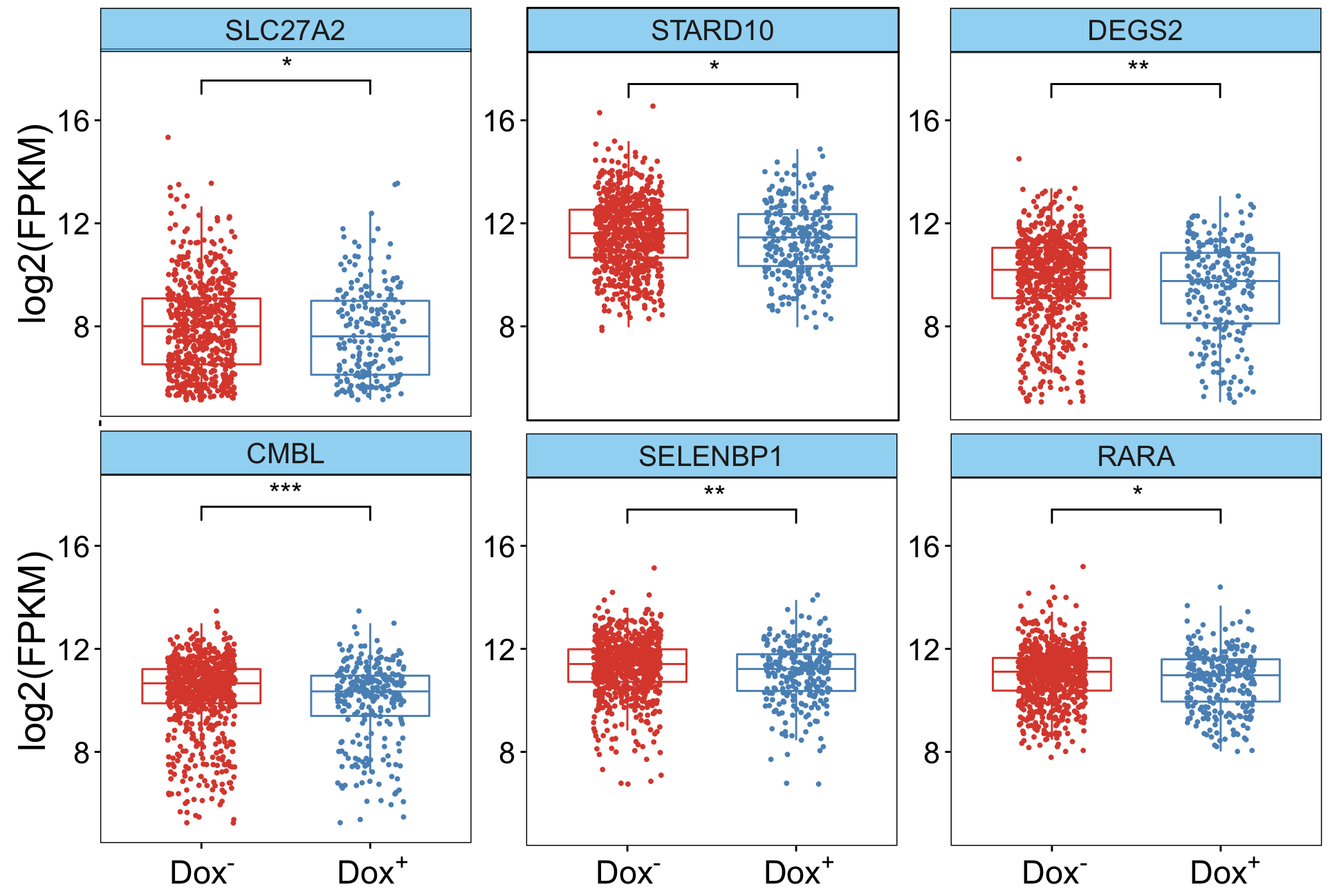


**Supplementary Figure S5. Analysis of gene expression for breast cancer patients from TCGA datasets.**

Box plots showing the mRNA expression levels (FPKM) of critical DEGs in Figure S4 in Dox+ and Dox- breast cancer subgroups derived from TCGA datasets. The P values were calculated by Wilcoxon’s signed-rank test. * P < 0.05, ** P < 0.01, *** P < 0.001.


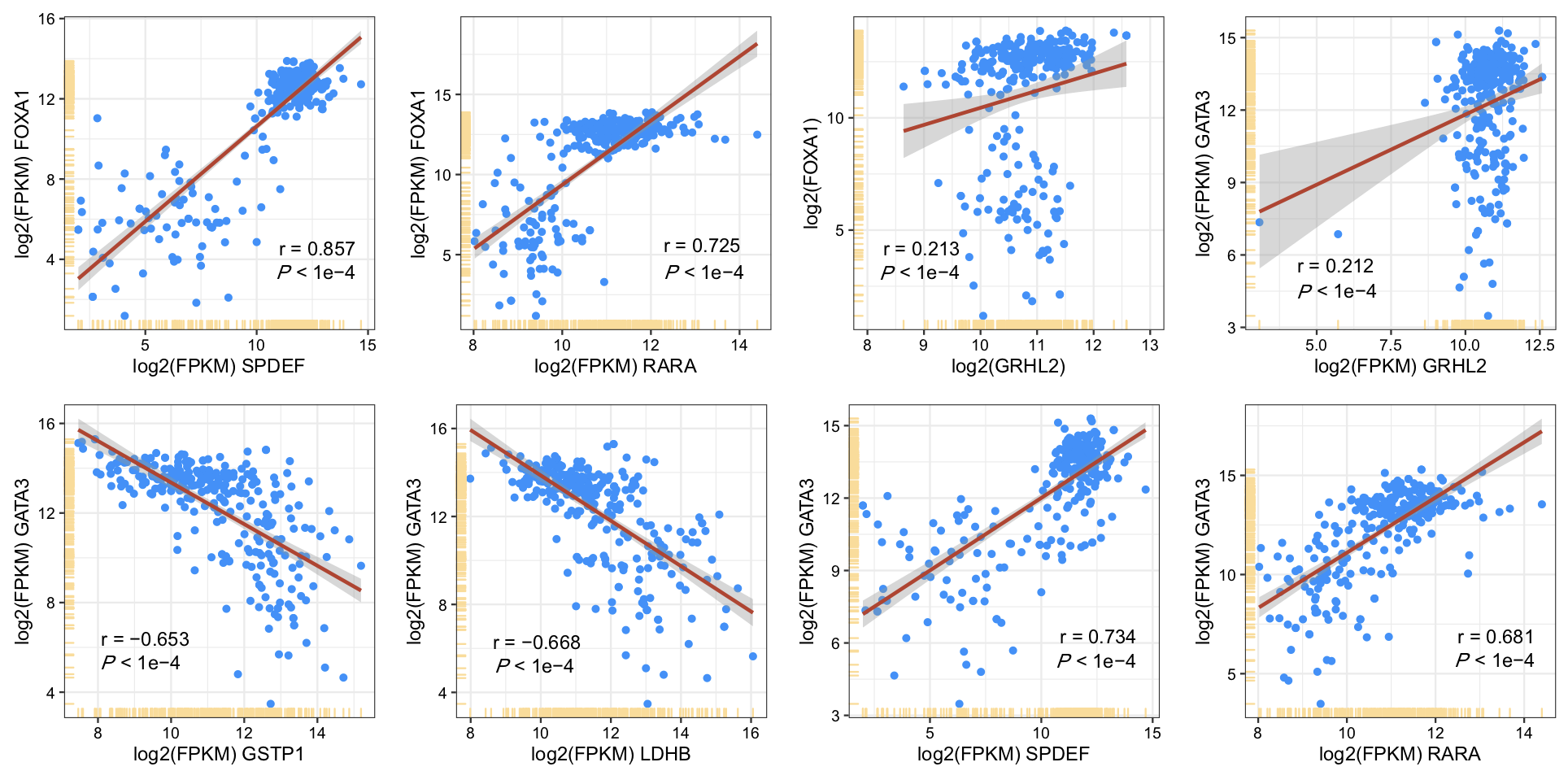


**Supplementary Figure S6. Gene correlation analysis for breast cancer patients from TCGA datasets.**

Correlations analysis of mRNA expression levels among critical DEGs in Figure S4 in the Dox+ breast cancer subgroup. The Pearson’s correlation coefficients (r) and P values were calculated using R packages. The P value less than 0.05 was considered to be statistically significant.

## Supplementary Tables

**Supplementary Table S1. FPKM of all genes.**

**Supplementary Table S2. FPKM of differentially expressed genes (DEGs).**

**Supplementary Table S3. ATAC-seq peaks identified by MACS2.**

**Supplementary Table S4. Annotation of differentially accessible regions (DARs).**

**Supplementary Table S5. Known TF binding motifs enriched in DARs.**
